# Temporal trends and global burden of ischemic heart disease attributable to non-optimal temperatures from 1990 to 2021: an analysis of the Global Burden of Disease Study 2021

**DOI:** 10.3389/fpubh.2025.1626504

**Published:** 2025-11-27

**Authors:** Li Chen, Yan Jiang, Jingyuan Wang, Zhenxun Wan, Ting Peng, Gang Luo, Qiuyu Liu, Mengnan Liu

**Affiliations:** 1 Affiliated Traditional Chinese Medicine Hospital, Southwest Medical University, Luzhou, China; 2 Department of Pediatrics, Southwest Medical University, Luzhou, China; 3 School of Pharmacy, Southwest Medical University, Luzhou, China

**Keywords:** Global Burden of Disease, non-optimal temperature, ischemic heart disease, climate change, time trend

## Abstract

**Background:**

Global climate change has intensified non-optimal temperature impacts on cardiovascular health. Ischemic heart disease (IHD), a leading cause of mortality, is increasingly linked to temperature anomalies driven by climate change, yet their association remains underexplored. Using Global Burden of Disease (GBD) 2021 data, this study analyzes spatiotemporal trends in temperature-attributable IHD burden from 1990 to 2021.

**Methods:**

GBD 2021 data on IHD mortality and disability-adjusted life years (DALYs) across 204 countries were analyzed via joinpoint regression. Stratified analyses by age, sex, region, and sociodemographic index (SDI) assessed subpopulation disparities.

**Results:**

In 2021, non-optimal temperatures were responsible for an absolute burden of 610,000 IHD deaths (95% UI, 459, 000 to 862, 000) and 1.24 million DALYs (95% UI, 915,900 to 1.76 million) globally. While these absolute figures represented increases of 41.8 and 37.8% since 1990, the age-standardized mortality rate (ASMR) and DALY rate (ASDR) actually decreased by 3.18 and 55.91%, respectively, over the same period, indicating that population growth and aging are key drivers of the rising absolute count. When examining the attribution of temperatures, the impact of low temperatures (accounting for 81.8% of the burden) was significantly higher than that of high temperatures (accounting for 18.2% of the burden). Regional disparities persisted, with high-SDI regions experiencing the largest decline in ASMR (6.5%), while low/middle-SDI regions faced rising burdens. The impact of high temperatures grew faster in these vulnerable regions. Males had higher absolute deaths and ASMR than females, and population of older adults were most vulnerable.

**Conclusion:**

Non-optimal temperatures, particularly rising high-temperature impacts, are critical environmental risks for IHD. The accelerating high-temperature-attributable ASMR over the past decade highlights underestimated risks. Region-specific strategies addressing climatic and demographic vulnerabilities are urgently needed to mitigate future IHD burdens.

## Introduction

IHD, as one of the leading global causes of morbidity and mortality, exhibits a disease burden intricately linked to diverse environmental and socioeconomic factors (1). According to the 2021 GBD, IHD has remained the foremost cause of death worldwide over the past three decades, resulting in nearly 9 million deaths in 2021 and contributing to 16% of global total mortality (2). With the advancements of medical technology and public health interventions have driven a decline in IHD ASMR in high-income countries (3). However, the burden remains persistently elevated in low- and middle-income nations, particularly those impacted by rapid urbanization, air pollution, and lifestyle transitions (4). In recent years, the escalating frequency of extreme temperature events induced by climate change has heightened global attention toward the influence of environmental temperature on cardiovascular health (5). Non-optimal temperature, defined as exposures to temperatures deviating from the human thermoneutral zone (including both heat and cold), has been progressively quantified in GBD studies as a contributor to disease burden (6). Epidemiological evidence indicates that temperature anomalies constitute a major risk factor for cardiovascular diseases, with strong associations observed for cause-specific mortality and morbidity (7, 8). Both heat and cold may trigger acute IHD events through direct or indirect mechanisms such as increased blood viscosity, blood pressure fluctuations, and exacerbated inflammatory responses (9). However, existing research has predominantly focused on the short-term health impacts of singular extreme temperature events (e.g., heatwaves or cold spells) (10), leaving a critical gap in systematic analyses of long-term temporal trends and regional heterogeneity in IHD burden attributable to varied temperature exposures (heat, cold, and non-optimal temperatures) (11).

GBD database serves as an authoritative data foundation for evaluating spatiotemporal variations in health impacts attributable to diverse risk factors (12); however its analyses of temperature-IHD associations have been predominantly confined to specific years or geographical regions with insufficient interpretation of dynamic trends across temporal intervals (13, 14). Leveraging GBD 2021 data this study presents the first systematic assessment of global regional and national-level temporal trends in IHD mortality and DALYs attributable to non-optimal temperatures while examining how population aging climate adaptation capacity and socioeconomic disparities influence disease burden distribution. The cumulative long-term effects of temperature-related IHD burdens are further elucidated through longitudinal analysis. These findings not only address critical gaps in understanding protracted temporal patterns of temperature-associated IHD but also deliver essential evidence to inform climate-resilient public health policy formulation where epidemiological insights into thermal exposure impacts are required. The analytical framework developed demonstrates how temperature-related health burdens may be modulated by demographic transitions and adaptive capacities which could guide targeted interventions for vulnerable populations. It should be emphasized that the quantified disease burden estimates reflect interactions between environmental exposures and population vulnerability factors that were rigorously analyzed through advanced statistical modeling techniques.

## Materials and methods

### Study data

GBD database represents the most comprehensive epidemiological surveillance system globally and has been extensively utilized in health policy formulation and disease burden reporting across numerous studies. It estimates health losses attributable to premature mortality and non-fatal disability by quantifying disease burdens from 87 risk factors, 369 diseases and injuries, and 286 causes of death across 204 countries and territories. The risk factors encompass metabolic, behavioral, and environmental determinants (2). This investigation extracted mortality data, DALYs, ASMR, and ASDR related to IHD attributable to high temperature(based on the minimum mortality temperature benchmark, the range of hot temperatures that lead to increased risk of death), low temperature(based on the minimum mortality temperature benchmark, the range of cold temperatures that lead to increased risk of death), and non-optimal temperature (the sum of health risks from high and low temperatures) exposure from the GBD database between 1990 and 2021^1^ (6, 15). SDI was employed to evaluate regional development levels through a composite metric integrating lag-distributed per capita income, average educational attainment among individuals aged ≥15 years, and total fertility rates for those under 25. Countries and territories were categorized into quintiles based on SDI values (ranging geometrically from 0 to 1): low (<0.46), low-middle (0.46–0.60), middle (0.61–0.69), high-middle (0.70–0.81), and high (>0.81), with elevated values indicating superior socio-demographic development (16). The methodology adheres to the Guidelines for Accurate and Transparent Health Estimates Reporting (GATHER) (17), ensuring compliance with international standards for epidemiological reporting.

The temperature estimates are derived from the gridded reanalysis dataset generated by the European Centre for Medium-Range Weather Forecasts (ECMWF) which features 0.25° × 0.25° spatial resolution and sub-daily temporal resolution (18). This high-resolution dataset provides the foundational exposure data required for assessing temperature-related health risks. Building upon this data, the burden of IHD attributable to high, low, and non-optimal temperatures is estimated through comprehensive methodological approaches adopted in the GBD study. For the estimation of temperature-related IHD burden, the present analysis adhered to the established methodological framework of the GBD Study 2019 (15). This framework integrates high-resolution temperature exposure data with exposure-response relationships between temperature and IHD. The foundational exposure-response relationships were derived from specific regions with available epidemiological data, such as North America, Europe, and East Asia. To generalize these relationships across diverse geographical locations globally, the GBD framework employed a meta-regression framework—Meta-Regression Bayesian Regularized Trimmed Tool (MR-BRT)—and comparative risk assessment metrics (16). By incorporating country-level covariates, including the Socio-demographic Index, average temperature, and healthcare access, the core exposure-response curves were adapted and refined (16). This process is predicated on the key assumption that while the overall shape of the temperature-health relationship is similar across regions, its magnitude may be modified by the aforementioned macro-level covariates. In this study, the regionally adjusted exposure-response curves were combined with daily gridded temperature data for each unit, enabling the calculation of the IHD burden attributable to high temperature, low temperature, and non-optimal temperature.

### Statistical analysis

This study employed Joinpoint regression models to estimate the uncertainty intervals (95% UI) for deaths, DALYs, ASMR, and ASDR attributable to non-optimal temperature-related IHD, which were demonstrated to depend on categorical or hierarchical variables, and the result of analysis were addressed from GBD’s original models. The 95% UI represents the 2.5th and 97.5th percentiles of 1,000 draw-level estimates generated in this study for each parameter. Age-standardized rates were applied using the GBD global reference population to enable comparisons across regions and periods. Two hundred four countries and territories were categorized into 21 GBD regions based on geographical proximity and stratified into five SDI regions according to their sociodemographic index levels. The global, regional, and national burdens of temperature-variation-induced IHD from 1990 to 2021 were systematically estimated using publicly accessible GBD 2021 data (GBD Cause of Death Summaries; ICD-10 codes I20-I25). Percentage changes and average annual percentage changes (AAPCs) were utilized to assess temporal trends during this 32-year period. The AAPCs were derived in this study through Joinpoint regression modeling (Version 4.9.1.0; National Cancer Institute in Rockville, Maryland, USA) which incorporates segmented linear statistical models to objectively evaluate temporal patterns in disease burden (19, 20). This methodology was designed to overcome the inherent subjectivity of conventional linear trend analyses through implementation of least squares regression for identifying inflection points in incidence variations. The model computes the sum of squared residuals between estimated and observed values to determine optimal transition points in temporal trends.

In this study, the AAPCs and their 95% confidence intervals (CIs) for ASMR or ASDR were calculated through segmented linear regression analysis where y represents ASMR or ASDR, i denotes the slope coefficient for each segment within the projected timeframe, and w_i_ indicates the length of each segment. The number of joinpoints was determined through permutation tests with a default maximum of three (21). Trends were classified as increasing if both AAPCs and corresponding 95% CIs exceeded zero whereas decreasing trends were defined by AAPCs with 95% CIs below zero. Otherwise, ASMR and ASDR were considered stable. Subsequent analyses illustrated temporal patterns in IHD burden attributable to temperature variations across different SDI regions and thermal conditions. The associations between 2021 AAPCs and country-level ASMR, ASDR, and SDI were further explored and analyzed using scatterplot smoothing techniques. Stratified analyses were conducted to identify vulnerable populations affected by high, low, and non-optimal temperatures in IHD outcomes, with subgroup evaluations based on age, sex, and SDI regional classifications. All statistical procedures were implemented in R programming language (version 4.3.2; R Core Team) using key packages including segmented (v1.6–4) for joinpoint regression and ggplot2 (v3.4.4) for visualization, with methodological rigor maintained through adherence to the Global Burden of Disease 2021 analytical framework. Notably, trends exhibiting AAPCs with 95% CIs spanning zero were interpreted as demonstrating stability rather than directional changes.

## Results

### The temporal trends in the global burden of IHD attributable to non-optimal temperatures from 1990 to 2021

As presented in Table 1, the global mortality attributable to non-optimal temperatures (encompassing both heat and cold exposure) for IHD exhibited an increase from 355,000 deaths (95% UI, 291,000 to 465,000) to 610,000 deaths (95% UI, 459,000 to 862,000) between 1990 and 2021, representing a 41.8% elevation. Simultaneously, DALYs associated with temperature-related IHD demonstrated growth from 772, 100 (95% UI, 622, 800 to 1, 015, 700) to 1, 241, 600 (95% UI, 915, 900 to 1, 764, 000), corresponding to a 37.8% increment. The substantially larger decrease in ASDR likely reflects improvements in survival leading to longer lifespans with potential disability, alongside advancements in managing non-fatal IHD outcomes. The ASMR and age-standardized ASDR displayed declines of 3.18 and 55.91%, respectively, during this period. This epidemiological pattern indicates that demographic aging processes combined with population expansion have been recognized as predominant contributors to the observed escalation in absolute disease burden.

**Table 1:** The deaths and ASMR of IHD attributed to non-optimal temperatures in 1990 and 2021, and its temporal trends from 1990 to 2021.

Characteristics	1990		2021		1990–2021 AAPCs No.(95% CI)
	Deaths No. × 10^3^ (95% UI)	ASMR No. (95% UI)	Deaths No. × 10^3^ (95% UI)	ASMR No. (95% UI)	
Global	355.69 (291.4, 465.06)	10.57 (8.63, 13.74)	610.52 (459.42, 862.75)	7.39 (5.57, 10.44)	−1.20 (−2.07, −0.33)
Sex					
Female	170.01 (137.75, 220.71)	8.95 (7.26, 11.57)	270.73 (203.91, 380.65)	5.79 (4.35, 8.14)	−1.46 (−2.30, −0.61)
Male	185.68 (150.37, 244.73)	12.50 (10.22, 16.41)	339.80 (255.30, 473.53)	9.33 (7.05, 12.98)	−0.99 (−1.88, −0.09)
Temperature					
High	32.57 (−0.68, 86.63)	0.90 (−0.03, 2.46)	112.40 (17.05, 256.43)	1.34 (0.20, 3.07)	1.29 (−1.61, 4.27)
Low	325.85 (285.69, 387.32)	9.74 (8.52, 11.60)	505.30 (432.02, 619.92)	6.14 (5.23, 7.53)	−1.48 (−2.42, −0.54)
SDI level					
Low SDI	12.35 (7.84, 17.92)	6.35 (4.02, 9.13)	27.32 (17.63, 38.69)	6.41 (4.17, 9.00)	0.04 (−1.78, 1.89)
Low-middle SDI	47.96 (28.87, 68.70)	9.00 (5.48, 12.90)	124.31 (74.28, 186.37)	9.53 (5.74, 14.23)	0.23 (−1.47, 1.95)
Middle SDI	65.83 (48.10, 88.91)	8.06 (5.83, 10.80)	184.32 (137.99, 251.71)	7.85 (5.93, 10.74)	−0.10(−1.00, 0.81)
High-middle SDI	111.04 (96.12, 141.41)	13.43 (11.58, 17.12)	241.84 (140.20, 241.84)	9.07 (7.30, 12.61)	−1.43 (−2.41, −0.43)
High SDI	118.03 (100.41, 154.88)	10.75 (9.12, 14.12)	99.83 (79.89, 136.20)	4.25 (3.43, 5.82)	−3.05 (−4.18, −1.91)
Super region					
Central Europe, eastern Europe, and central Asia	84.40 (75.18, 108.44)	20.20 (17.88, 25.98)	100.73 (83.99, 137.26)	15.27 (12.73, 20.81)	−0.98(−2.13, 0.18)
Latin American and Caribbean	9.29 (7.51, 11.45)	4.85 (3.90, 5.95)	19.15 (16.19, 22.87)	3.19 (2.69, 3.81)	−1.42 (−3.26, 0.46)
North Africa and Middle East	35.32 (24.16, 50.52)	5.26 (17.37, 35.90)	73.54 (49.93, 111.85)	19.25 (13.23, 28.87)	−0.83 (−2.15, 0.50)
Southeast Asia, east Asia, and Oceania	49.22 (37.26, 66.45)	5.8 (4.35, 7.90)	168.791 (127.25, 234.43)	7.17 (5.41, 10.03)	0.55 (−0.60, 1.72)
Sub-Saharan Africa	5.47 (3.09, 7.81)	3.18 (1.79, 4.58)	11.63 (8.55, 15.70)	3.07 (2.28, 4.19)	−0.11 (−1.57, 1.38)
South Asia	49.03 (26.28, 71.94)	9.56 (5.08, 14.12)	144.16 (78.93, 211.40)	10.70 (5.88, 15.64)	0.33 (−1.99, 2.71)
High income	111.04 (96.12, 141.41)	13.43 (11.58, 17.12)	92.53 (73.74, 119.99)	3.62 (2.95, 4.70)	−3.40 (−4.48, −2.31)

No., number; UI, uncertainty interval; CI, confidential interval; ASMR, Age-standardized mortality rate (per 100,000); AAPCs, average annual percentage change; SDI, Socio-demographic index.

### Regional and sociodemographic index-stratified analyse

**Figure 1:**
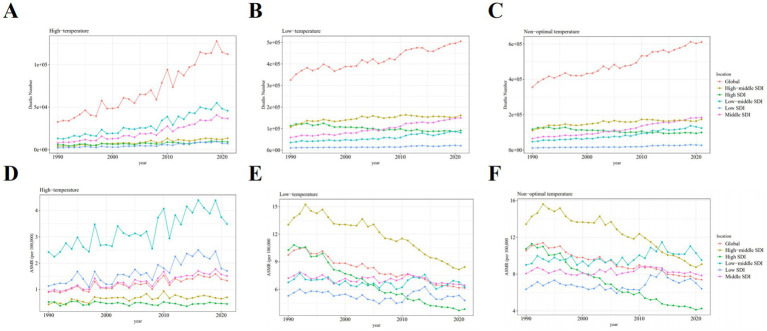
Trends in IHD deaths attributable to high, low, and non-optimal temperatures globally and across SDI-specific regions from 1990 to 2019. **(A,D)** High temperature. **(B,E)** Low temperature. **(C,F)** Non-optimal temperature. ASMR, age-standardized mortality rate. SDI, sociodemographic index.

**Figure 2:**
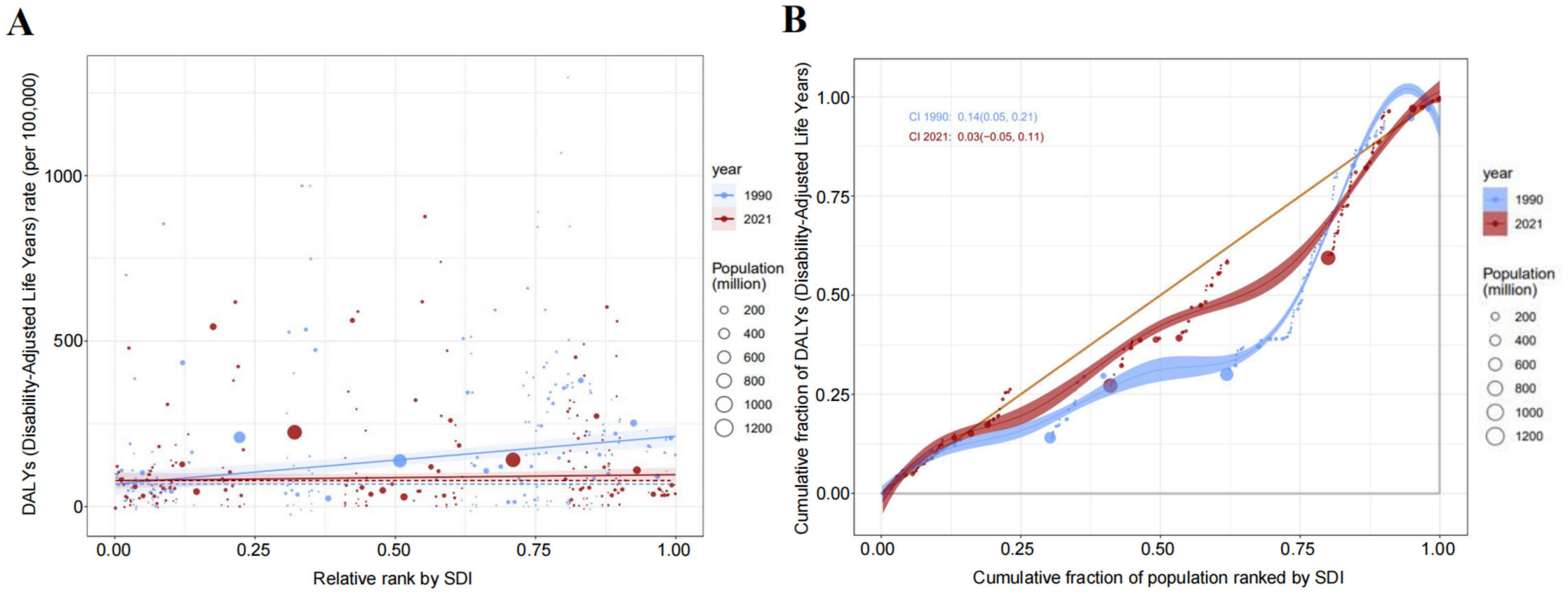
Health inequality analysis. Regression lines presenting income-related health inequalities in the burden of IHD attributable to non-optimal temperatures from 1990 to 2021. **(A)** Absolute inequality analysis; **(B)** Relative inequality analysis.

**Figure 3:**
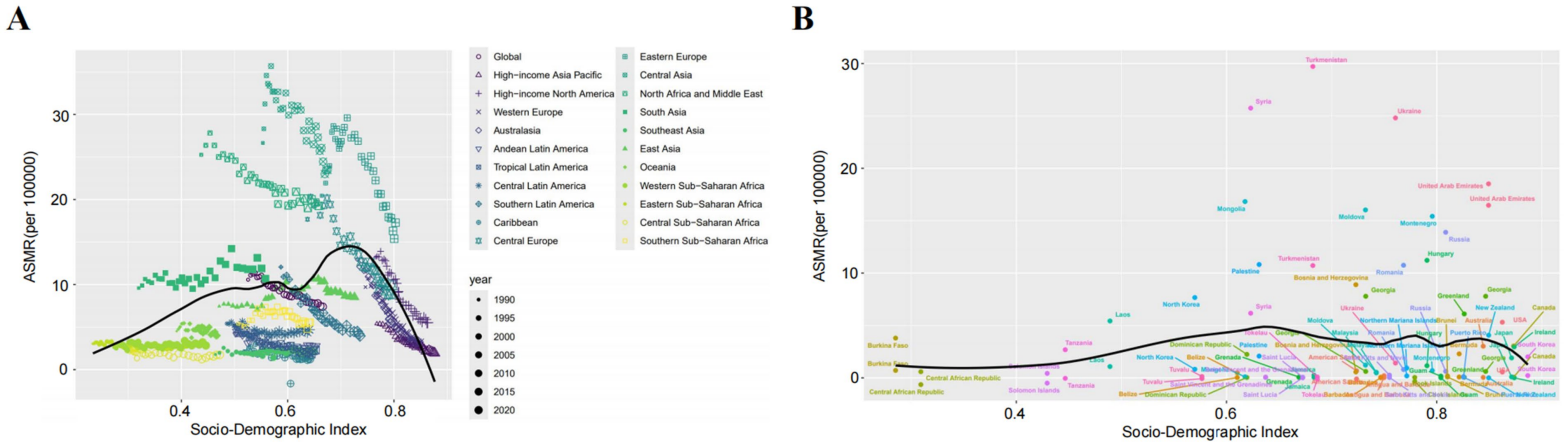
**(A)** Relationship between SDI and ASMR for IHD attributable to non-optimal temperatures across 22 global regions. **(B)** Relationship between SDI and ASMR for IHD attributable to non-optimal temperatures across 204 countries worldwide. Areas above the curve indicate a higher-than-expected burden, while areas below the curve indicate a lower-than-expected burden.

**Figure 4:**
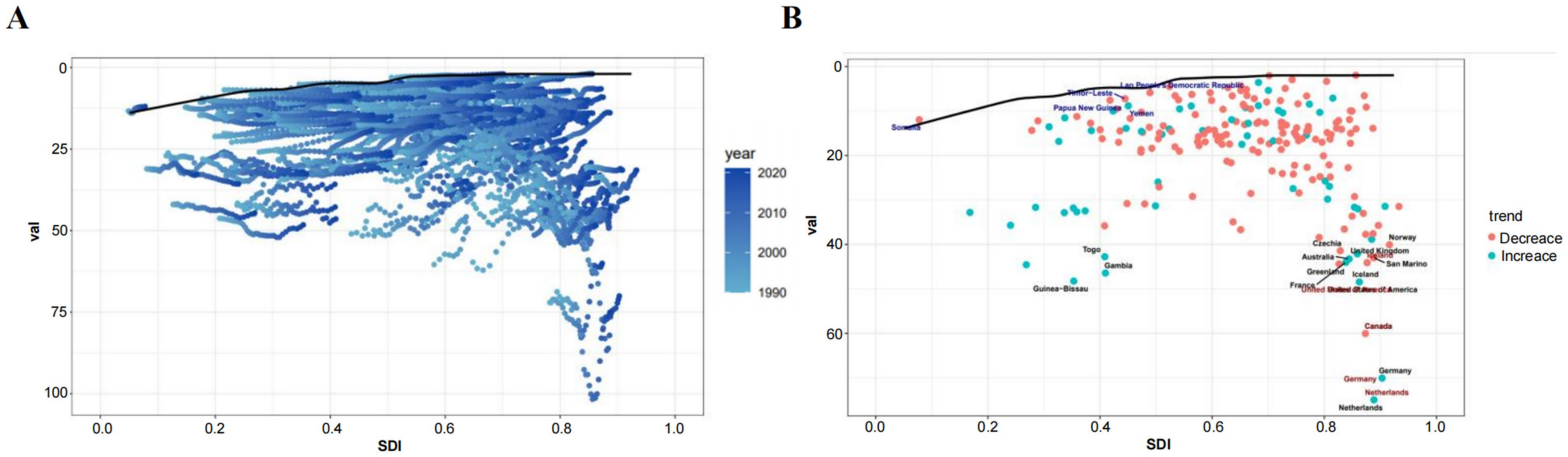
Frontier analysis of age-standardized IHD DALYs rates attributable to non-optimal temperature based on SDI in 2021. **(A)** The frontier is demarcated by a solid black line, with countries and regions indicated by circular dots. **(B)** Blue markers denote frontier countries with low SDI (<0.5) and low effective disparities (e.g., Somalia, Yemen, Laos), while red markers represent countries and regions with high SDI (>0.85) and relatively higher developmental-level effective disparities (e.g., Netherlands, Canada, United States). Red dots indicate increases in non-optimal temperature-related IHD DALYs rates from 1990 to 2021. Blue dots represent decreases in age-standardized non-optimal temperature-related IHD DALYs rates during the same period 1990–2021.

IHD attributable to non-optimal temperatures exhibited substantial geographical heterogeneity between 1990 and 2021. Among the five SDI regions, mortality counts demonstrated divergent trends with high-SDI countries experiencing declines while the remaining four regions showed increases (Table 1; Figures 1A–C). Notably, low- and low-middle SDI nations displayed pronounced mortality growth under high-temperature conditions. ASMR revealed distinct temporal patterns across SDI quintiles (Table 1; Figures 1D–F). Low-SDI countries exhibited marginal ASMR increases from 6.35% (95% UI, 4.02–9.13) to 6.41% (95% UI, 4.17–9.00) with AAPC 0.04 (95% CI, −1.78–1.89), while low-middle SDI regions showed more marked elevation from 9.00% (5.48–12.90) to 9.53% (5.74–14.23) with AAPC 0.23 (−1.47–1.95). The absolute mortality surge of 64.1% in low-middle SDI countries substantially exceeded global averages. Contrastingly, middle-, high-middle-, and high-SDI regions demonstrated declining trends: middle SDI [8.06% (5.83–10.80) to 7.85% (5.93–10.74); AAPC −0.10 (−1.47–1.95)], high-middle SDI [13.43% (11.58–17.12) to 9.07% (7.30–12.61); AAPC −1.43 (−2.41–0.43)], and high SDI [10.75% (9.12–14.12) to 4.25% (3.43–5.82); AAPC −3.05 (−4.18–1.91)]. The health inequality analysis results demonstrated in Figure 2 reveal that the concentration index (CII) (quantify indicators of the concentration of health burdens associated with socioeconomic status) of (DALYs) per 100,000 population attributable to non-optimal temperatures was 0.14 (0.05, 0.21) in 1990 and 0.03 (−0.05, 0.11) in 2021, indicating that the disparity in IHD burden between high-income and low-income countries was substantially reduced throughout the 1990–2021 observation period. This progressive narrowing of health inequality gap was evidenced by the CII approaching statistical neutrality over the three decades, suggesting improved equity in temperature-related cardiovascular health outcomes across nations with varying socioeconomic statuses. At the super-regional level in 2021, Central-Eastern Europe and Central Asia recorded the highest ASMR whereas sub-Saharan Africa and Latin America showed the lowest (Figure 3A). South Asia, Southeast Asia, East Asia, and Oceania were identified as regions with rising AAPC trends (Table 1). Frontier analyses delineated the evolving relationship between SDI and ASDR across development levels (Figures 3B, 4A,B). An inverse pattern emerged where high-SDI (>0.85) countries like Canada and Norway experienced ASDR increases while low-SDI (<0.50) nations such as Gambia and Togo demonstrated reductions. These findings suggest greater potential for burden mitigation in higher-SDI regions despite their current epidemiological advantages. The data collectively underscore critical interactions between socioeconomic development, environmental exposures, and cardiovascular health outcomes. The widening health disparities and divergent mortality trends emphasize the necessity for context-specific public health interventions that account for regional climate vulnerabilities and healthcare infrastructure capacities.

### IHD burden attributable to non-optimal temperature by age and gender

**Figure 5:**
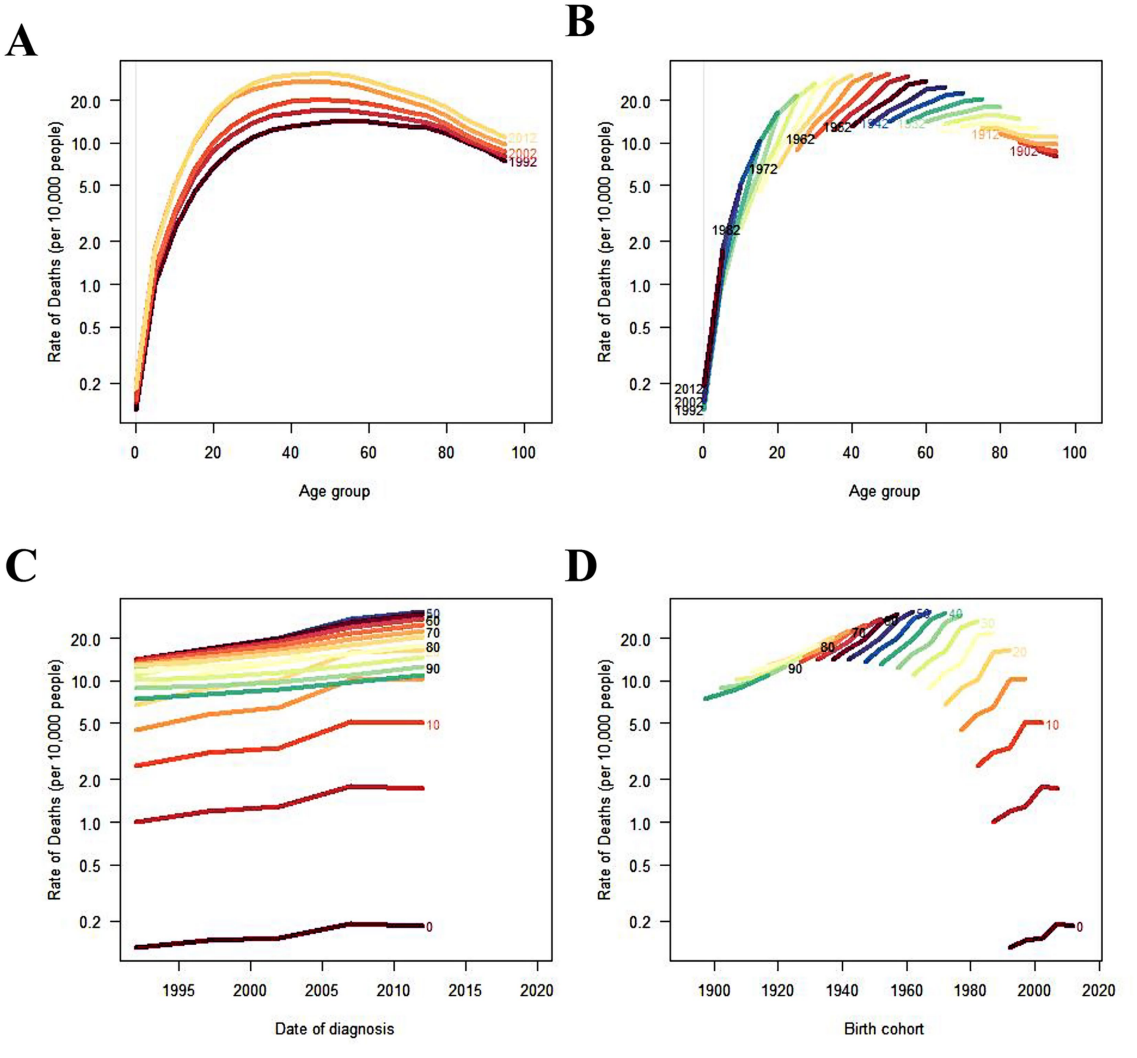
Trends of age-specific, period-based and cohort-based variation of IHD mortality attributable to non-optimal temperatures globally. **(A,B)** IHD mortality across different age groups. **(C)** Period-based IHD mortality rates. **(D)** Cohort-based IHD mortality rates.

**Figure 6:**
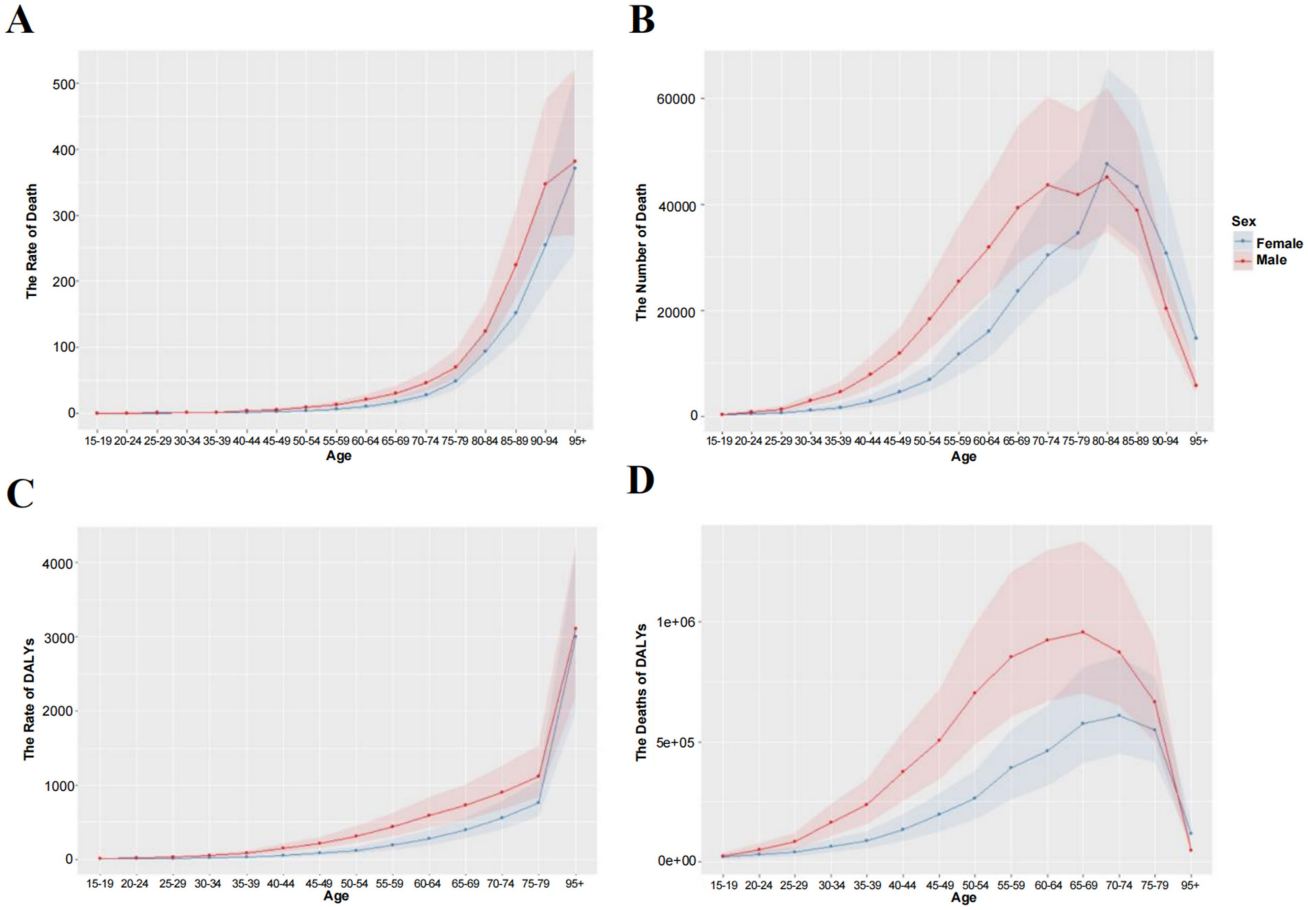
Line chart. Global age-specific mortality and deaths from IHD attributable to non-optimal temperatures in 2021. **(A)** Age-specific mortality rate. **(B)** Age-specific death count. **(C)** Number of DALYs. **(D)** DALY rate.

**Figure 7:**
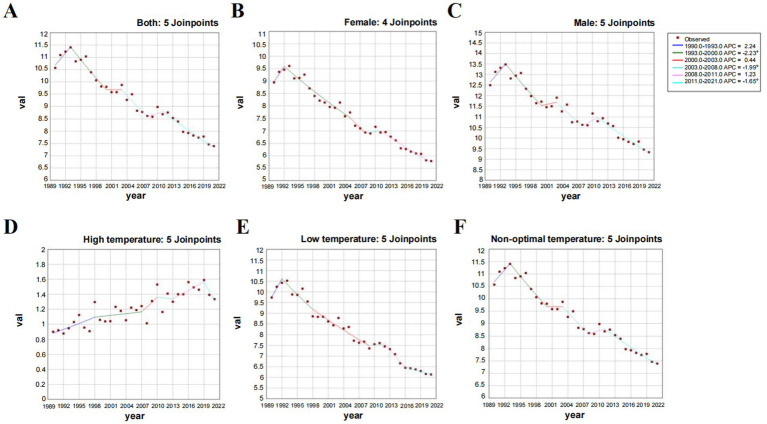
**(A–C)** Joinpoint regression analysis of global age-standardized mortality rates from IHD attributed to non-optimal temperatures by gender (1990–2021): **(A)** Overall. **(B)** Female. **(C)** Male. **(D–F)** Joinpoint regression analysis of global age-standardized mortality rates from IHD attributed to high temperature, low temperature, and non-optimal temperatures (1990–2021): **(D)** High temperature. **(E)** Low temperature. **(F)** Non-optimal temperatures.

Figure 5 demonstrates the age-period-cohort effects of global IHD deaths attributable to non-optimal temperatures, indicating that IHD mortality rates initially increase with age before subsequently declining. As illustrated in Figure 6 the mortality counts for males and females exhibited progressive escalation across age strata reaching peak values in the 85–89 age group. A notable mortality reduction was observed specifically in males aged 75–79 years. The ASMR displayed exponential growth with advancing age while maintaining consistently higher values in males compared to females (Figures 6A,B). Regarding DALYs the maximum burden occurred earlier in the 65–69 age group for both genders with the age-standardized ASDR demonstrating an incremental pattern associated with aging (Figures 6C,D). A critical observation reveals that beyond 79 years the ASDR for both sexes undergoes dramatic acceleration suggesting population of older adults bear the predominant burden of temperature-related IHD morbidity. Joinpoint regression analysis indicates an overall downward trend in ASMR for non-optimal temperature-attributable IHD from 1990 to 2021 with male AAPC estimated at −0.99 (95% CI, −1.88 to −0.09) and female AAPC at −1.46 (95% CI, −2.30 to −0.61). Despite these temporal reductions male subjects consistently exhibited higher absolute mortality counts and ASMR values throughout the observation period compared to their female counterparts (Figures 7A–C).

### Differences in IHD disease burden by temperature type

**Figure 8:**
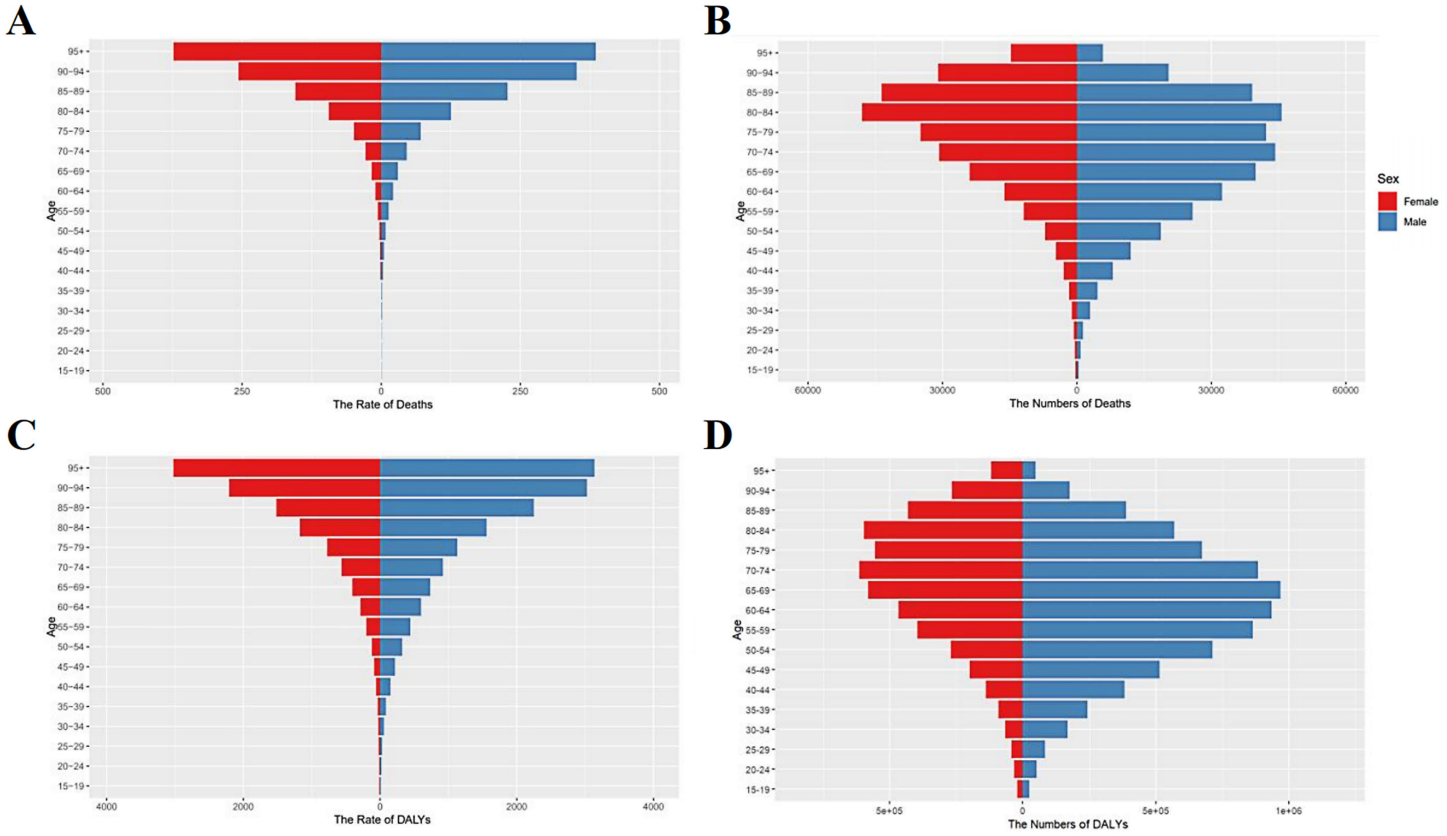
Dual-axis chart. Global burden count and proportion of IHD attributable to non-optimal temperatures by sex and age in 2021 **(A)** Death count. **(B)** Death rate. **(C)** DALYs number; **(D)** DALY rate.

As shown in Table 1, substantial increases in IHD deaths and DALYs attributable to high temperature, low temperature, and non-optimal temperature were observed between 1990 and 2019. The mortality and DALYs induced by low-temperature exposure consistently exceeded those associated with high temperatures throughout this period. Notably however, the ASMR related to high temperature demonstrated an accelerated upward trend during the past decade. At the SDI regional level, middle-low income countries exhibited more pronounced increases in both absolute high temperature-related IHD deaths and ASMR compared to middle-high income nations (Table 1; Figures 1A, 7D) with the disparities being particularly marked in mortality statistics. Although high-income countries maintained the highest absolute low temperature-attributable IHD mortality, the ASMR across all five SDI regions displayed consistent downward trajectories (Table 1; Figures 7E,F) indicating a progressive decline in the impact of low temperature on IHD disease burden. The epidemiological transition pattern reveals that middle-low income countries are experiencing disproportionately elevated climate-related cardiovascular risks particularly from heat exposure whereas cold-related burdens show gradual mitigation across socioeconomic strata. This divergence underscores the emerging public health challenge posed by global warming in vulnerable populations despite ongoing reductions in cold-associated mortality burden (see Figure 8).

## Discussion

This study, based on GBD data, comprehensively assessed the long-term effects and dynamic trends of non-optimal temperatures (i.e., exposure to environments that deviate from the optimal temperature range) on the burden of IHD globally between 1990 and Previous studies, such as Wei et al. (22), showed an upward trend in IHD due to non-optimal temperatures, but this study revealed that, over the past decade, age-standardized mortality rates associated with IHD caused by non-optimal temperatures have declined, although the burden remains significant. Non-optimal temperatures may exacerbate the global health inequality associated with cardiovascular diseases, thereby increasing the burden of IHD worldwide. The IHD-related deaths and DALYs caused by non-optimal temperatures account for 18.2% (95% UI, 14.6 to 21.8%) of the global burden, with considerable differences in trends and severity across regions and countries. Specifically, the death burden caused by low temperatures is significantly higher than that caused by high temperatures, which is consistent with previous research (23). However, this study found that the age-standardized mortality rate associated with high temperatures has accelerated over the past decade, which differs significantly from previous studies (24). Gasparrini et al.’s (7) team, based on multi-country data, found that the relative risk of cardiovascular disease due to low temperatures is higher than that due to high temperatures, but they did not specifically distinguish the specific impact of temperature types on IHD. This study, through integrating climate zoning models and GBD data, revealed the individual impacts of high, low, and non-optimal temperatures as independent risk factors on the burden of IHD. Moreover, this study extended the time-trend analysis to the period following the COVID-19 pandemic (2020–2021), revealing that the increase in IHD mortality associated with high temperatures in middle and low-income countries was 1.3 times that of 2010–2019, suggesting that the interaction between climate change and the vulnerability of health systems may exacerbate the global inequality of disease burden.

Most early studies primarily focused on the acute health effects of extreme temperature events while underestimating the cumulative impacts of persistent non-optimal temperature deviations below extreme threshold (25). This study revealed that non-extreme heat exposure (daily mean temperatures exceeding regional optimal thresholds without reaching extreme levels) accounted for 64.2% of heat-related IHD mortality burden with its annual growth rate surpassing that of extreme heat events. The long-term cumulative effects of non-optimal temperatures on IHD have been substantially underestimated which challenges conventional public health strategies centered on extreme events and highlights the necessity to shift prevention priorities toward continuous temperature deviation monitoring. A pronounced gender disparity in temperature-related IHD mortality burden was identified with males exhibiting higher vulnerability than females across all thermal exposures (heat cold and non-optimal temperatures). From 1990 to 2021 the annual average percentage change in ASMR of cardiovascular diseases demonstrated greater decline among females across all age groups aligning with previous observations (26). These differential outcomes may stem from variations in thermoregulatory capacity physiological responses and socioeconomic or cultural determinants as hypothesized by researchers (24). Population of older adults showed heightened susceptibility to non-optimal temperature exposure compared to younger adults consistent with findings from Chen et al.’s (27) research. Notably the population of people over 80 years old exhibited significantly higher mortality among females than males warranting further investigation through prospective studies. The temperature-related IHD burden displayed substantial heterogeneity across SDI regions with high-SDI areas demonstrating greater absolute mortality despite middle-income countries showing escalating annual death counts particularly in heat-attributable IHD mortality within low-middle SDI nations. Investigations by Guo et al. (28). Romanello et al. (29) suggested that most low-to-middle SDI countries situated in tropical/subtropical zones experience intensified heat impacts compounded by occupational exposures in agriculture and construction sectors prevalent among their populations. Geographically Southeast Asia Eastern Europe and Sub-Saharan Africa bore heavier burdens from extreme temperature exposures whereas South Asia’s substantial population base resulted in the highest absolute burden. Frontier analyses indicated marked disparities in 2021 DALYs burden across SDI regions with higher-SDI areas demonstrating greater potential for burden reduction through improved healthcare accessibility. This threshold effect of medical resource availability on temperature-related health risks provides empirical justification for prioritizing primary healthcare system enhancements in low-middle income countries (6).

While this study addresses the critical gap in investigating long-term trends of temperature-related IHD burden, several limitations warrant acknowledgment. (1) the spatiotemporal resolution of exposure assessment remains constrained (30). Although GBD 2021 employed 0.5° × 0.5° gridded climate data, microenvironmental temperature exposures at the individual level—including indoor occupational conditions and air conditioning prevalence—were not fully captured, which might lead to underestimation of risks among high-risk occupational populations such as outdoor workers. (2) residual confounding effects persist despite the GBD model’s adjustment for covariates such as age, gender, and socioeconomic status. Other temperature-associated environmental covariates (e.g., air pollution and humidity) or behavioral factors (e.g., dietary patterns and physical activity levels) might not have been sufficiently controlled (31, 32), potentially compromising the reliability of causal inferences. (3) while the findings emphasize global and regional trends, they lack granular analysis of differential risk profiles among subnational vulnerable populations including aging communities and low-income groups (33, 34). Future research directions should prioritize the following aspects. (1) Climate-health integrated prediction models should be developed by synthesizing climate scenarios from the IPCC Sixth Assessment Report (AR6) with demographic projections to simulate spatiotemporal evolution patterns of IHD burden under diverse warming pathways (35). (2) The robustness of findings could be enhanced through systematic implementation of stratified analyses and multivariate regression models to mitigate confounding biases (36). (3) Regionally tailored interventions targeting special populations must be advanced, exemplified by pilot programs integrating heat-health early warning systems with primary care infrastructure. Such initiatives involving regional surveillance mechanisms and prospective health adaptation frameworks may facilitate targeted risk mitigation among vulnerable subgroups (29, 37). Targeted interventions—such as through targeted social care policies to protect the population of older adults, in order to enhance healthcare resilience and reduce temperature-related IHD mortality and disability rates, especially in severely affected regions and population groups.

## Conclusion

In summary, a significant association has been identified between non-optimal temperatures and IHD burden from 1990 to 2021. Globally, the burden of IHD attributable to non-optimal temperatures has exhibited complex temporal trends which pose a non-negligible threat to public health systems. Vulnerable populations particularly in low- and middle-income countries as well as older adults are disproportionately affected, with this environmental health challenge exacerbating existing strains on already overstretched healthcare resources and socioeconomic development in these regions. Future efforts must prioritize the translation of epidemiological evidence into actionable public health practices within an interdisciplinary framework to address the persistent threat posed by climate change to cardiovascular health. Multisectoral interventions should be urgently implemented to strengthen healthcare resilience and protect susceptible populations from temperature-related IHD mortality and disability.

## Data Availability

The datasets presented in this study can be found in online repositories. The names of the repository/repositories and accession number(s) can be found in the article/supplementary material.

## Footnotes

^1^ http://ghdx.healthdata.org/gbd-results-tool
